# Localized Pigmented Villonodular Synovitis in the Shoulder: Report of a Case Treated through Arthroscopy

**DOI:** 10.1055/s-0044-1779313

**Published:** 2024-04-22

**Authors:** Jair Simmer Filho, Thiago Henrique Cruz Ghidetti, Raul Meyer Kautsky

**Affiliations:** 1 Serviço de Ortopedia e Traumatologia, Hospital Santa Rita de Cássia, Vitória, ES, Brasil

**Keywords:** arthroscopy, giant cell tumor of tendon sheath, shoulder injuries, shoulder joint

## Abstract

Pigmented villonodular synovitis (PVNS) is rare in the shoulder, with few descriptions in the literature. We present the case of a 58-year-old female patient with no history of trauma. The patient reported pain for 2 months with no limb irradiation and presented lifting strength loss and progressive limitation of active and passive mobility. She underwent unsupervised physical therapy and there was no improvement in symptoms. A magnetic resonance imaging (MRI) scan of the shoulder showed an oval structure of approximately 2 cm in diameter in the anteroinferior region of the glenohumeral joint with internal hypointense signal foci. We performed the arthroscopic treatment, with marginal resection of the lesion and tenotomy of the long bicipital head. The anatomopathological report confirmed the diagnosis of PVNS. Four years and five months after the surgery, the patient is pain-free, with full recovery of the left shoulder function. A follow-up MRI showed no recurrence, demonstrating the effectiveness of the arthroscopic treatment in this case.

## Introduction

Pigmented villonodular synovitis (PVNS), also called tenosynovial giant cell tumor, is a proliferative disease affecting the synovial membrane in the joints, bursae, or tendon sheaths, which is associated with hemosiderin deposits. 1 It is a rare benign tumor, highly proliferative, and locally aggressive, resulting in joint destruction. 2 It has high recurrence rates, but malignant transformation is rare; 1 3 and the etiology is unknown, 2 potentially related to previous trauma and joint bleeding or rheumatoid arthritis (RA). 4 Differential diagnoses include other types of arthritis, such as RA, hemophilic arthropathy, tuberculosis, and neoplasms. 4 The prevalence is estimated at 1.8 cases per 1 million people, and the disease affects young adults between the third and fourth decades of life, with no gender preference, 5 6 7 but it may also affect children. 4

The condition is often mono-, intra-, or extra-articular, 1 and polyarticular involvement is exceptionally rare; 6 PVNS can manifest in a localized form (single tumor mass) or, more frequently, a diffuse form, affecting the joint compartment or the entire synovial membrane of the joint. 1 3 Shoulder involvement is very rare. 5 8 9 10 Mahieu et al. 8 found only 30 cases of shoulder PVNS in the French and English literature published until 2001, and they have estimated that the prevalence of shoulder PVNS is lower than 2%. 5 8 9

## Case Report

**Fig. 1:**
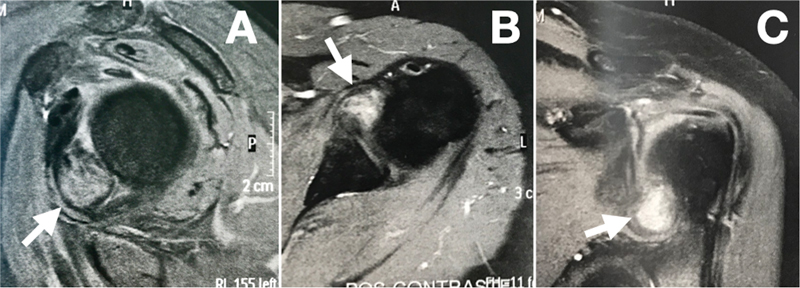
Magnetic resonance imaging. ( **A** ) The sagittal section shows an oval structure along the lower edge of the subscapularis tendon projecting into the glenohumeral joint (arrow). Note the lesion's hypodense sign. ( **B,C** ) Axial and coronal sections of the left shoulder respectively, obtained after the administration of a contrast agent, revealing an oval structure, with approximately 2 cm in diameter, in the anteroinferior region of the shoulder joint (arrows), with internal hypointense signal foci.

A 58-year-old female patient reported left shoulder pain for 2 months, lifting strength loss, and progressive worsening. She had no history of trauma or pain irradiation to the arm. The patient had undergone physical therapy with no symptom improvement. She had a history of fibromyalgia, anxiety disorder, and adhesive capsulitis on the contralateral shoulder. The physical examination revealed normal findings on ectoscopy, limited joint mobility only for lateral rotation (active range of motion in elevation, lateral rotation, and medial rotation of 150°, 70°, and T10 for the right shoulder, and of 150°, 50°, and T10 for the left shoulder respectively), grade-4 lifting strength (positive Jobe test), positive irritative maneuvers for subacromial impingement (positive Neer and Hawkins tests), and inflammation of the long head of the biceps tendon (positive Speed and Yergason tests). The radiographs were unremarkable. A magnetic resonance imaging (MRI) scan showed an oval structure with 2 cm in diameter in the anteroinferior region of the glenohumeral joint with internal foci of hypointense signal ( Fig. 1 ).

**Fig. 2:**
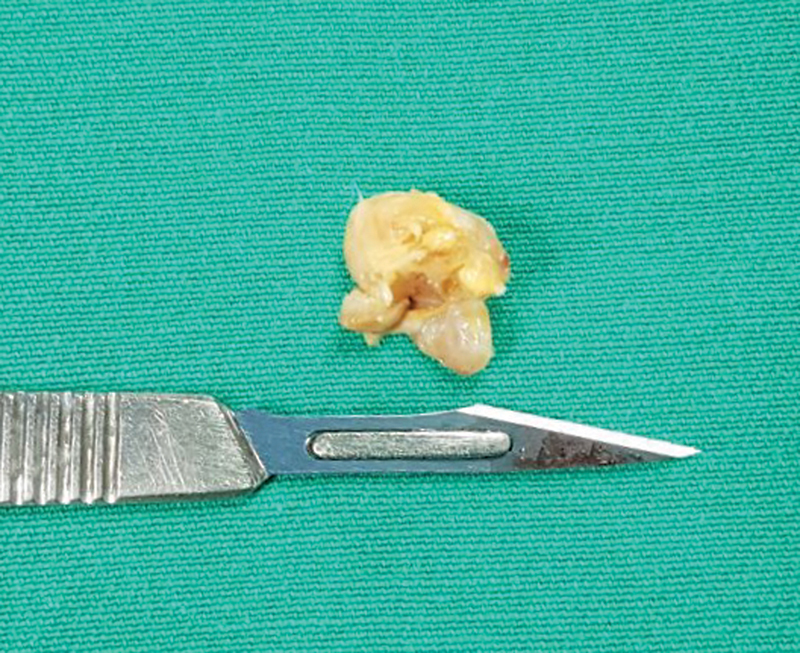
Nodular aspect with pigmentation by hemosiderin deposits, providing a yellowish-brown color. Surgical specimen removed by arthroscopic marginal resection.

**Fig. 3:**
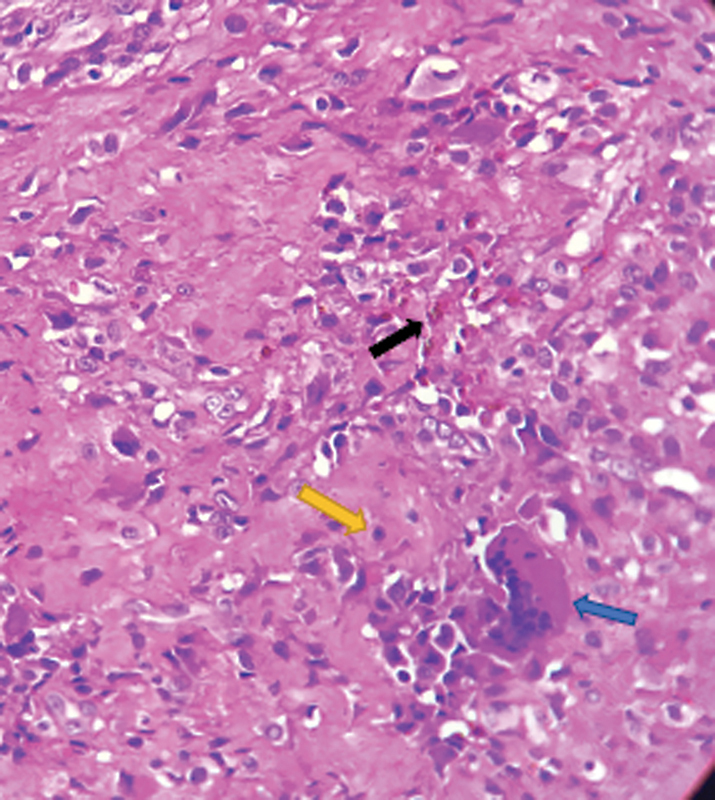
Pigmented villonodular synovitis consisting of round and oval cells with no atypia, histiocyte-like appearance (yellow arrow), and macrophages with hemosiderin deposits (black arrow) intermingled with giant cells (blue arrow) on the fibroconnective stroma. Abbreviation: HE, hematoxylin and eosin stain.

The treatment consisted of marginal resection of the lesion and arthroscopic tenotomy of the long head of the biceps in lateral decubitus. With the camera in the posterior portal (alternating between 30° and 70° optics), the resection was performed through the anterior portal, which was created slightly more medially to gain access to the humeral neck. During surgery, we identified a pedunculated, yellowish-brown, fibrotic, nodular mass in the anteroinferior region of the joint. The pedicle originated from the capsular attachment in the humeral neck, close to the axillary recess ( Fig. 2 ). The resection was performed with arthroscopic forceps, and the synovectomy in the axillary recess was performed without aspiration to avoid axillary nerve damage. We requested an anatomopathological examination of the surgical specimen to confirm the diagnosis of PVNS ( Fig. 3 ).

**Fig. 4:**
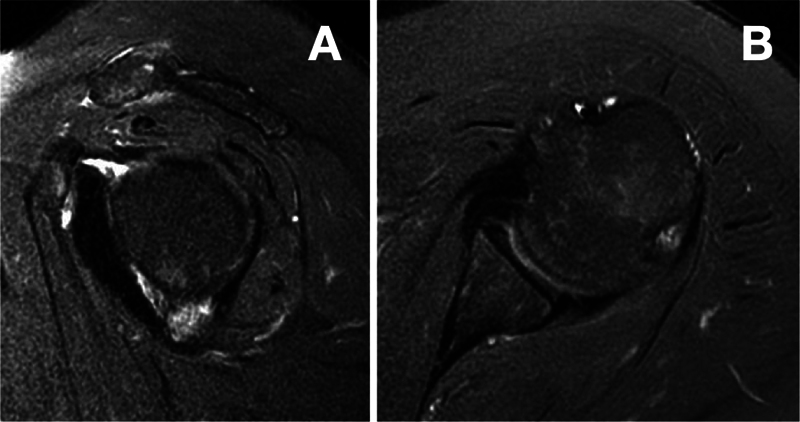
( **A,B** ) A follow-up MRI scan in sagittal and axial sections of the left shoulder one year and ten months after surgery revealing no images suggestive of disease recurrence.

**Fig. 5:**
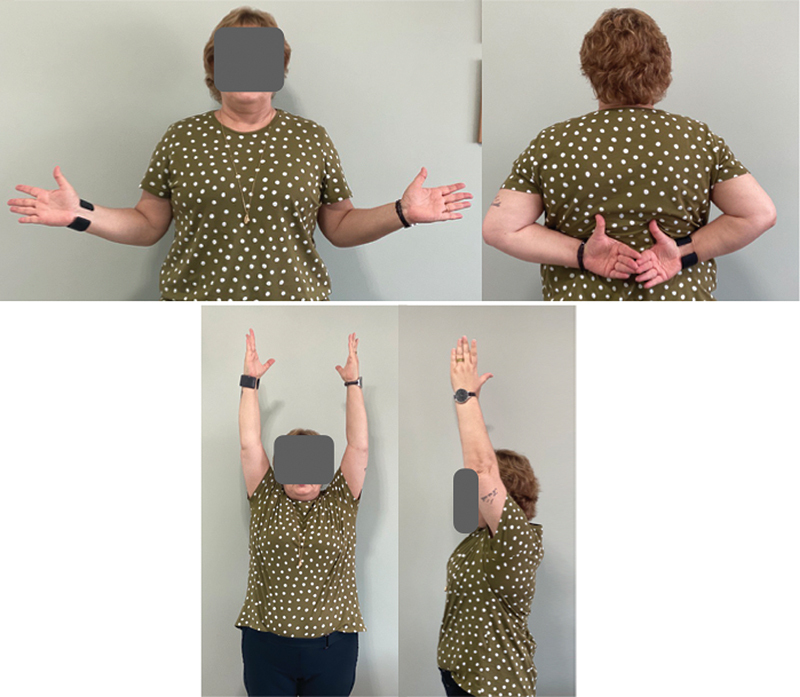
Postoperative clinical aspect four years and five months after surgery.

We did not prescribe adjuvant therapies. We started analgesic and motor physiotherapy in the first week after surgery. Initially, the patient had pain and joint stiffness that gradually improved over the following 10 months. The first postoperative follow-up MRI, performed 6 months after surgery, showed signs of capsular thickening consistent with adhesive capsulitis. The second MRI, 1 year and 9 months after surgery, did not show images suggestive of adhesive capsulitis or signs of neoplasm recurrence ( Fig. 4 ). Currently, 4 years and 5 months after surgery, the patient reports being asymptomatic, performing physical activity, and presenting full functional recovery in the operated (left) shoulder, with good active mobility (active range of motion in elevation, lateral rotation, and medial rotation of 150°, 70°, and T10 for the right shoulder, and of 150°, 60°, T10 for the left shoulder respectively) ( Fig. 5 ). She also presents grade-5 lifting strength with no pain (negative Jobe test), negative results for irritative maneuvers for subacromial impingement (negative Neer and Hawkins tests), and negative results for inflammation of the long head of the biceps tendon (negative Speed and Yergason tests).

The patient in the case herein reported gave her verbal and written consent authorizing the disclosure of her health data.

## Discussion

In the shoulder, PVNS is a rare, locally-invasive, and aggressive disease that may result in severe joint morbidity. Diagnosis requires correlating clinical, imaging, and histopathological findings. 3 Arthroscopic treatment is a minimally-invasive and effective alternative for the marginal resection of these lesions, especially in a case such as the one herein reported, in which the lesion was in the axillary recess.

The symptoms are nonspecific, making diagnosis a challenge. Arthralgia usually occurs with progressive antalgic limitation of movements, 9 11 joint stiffness, 11 erythema, joint effusion, and shoulder edema. 3 9 In the case herein reported, the initial hypothesis was of adhesive capsulitis due to the pain, stiffness on the baseline physical examination, and history of capsulitis of the contralateral shoulder. However, the MRI showed an image suggestive of localized PVNS, whose symptoms are often mechanical, 4 resulting from the interposition of the tumor mass in the joint space.

Extensive rotator cuff tears with osteoarthritis and bone erosion are frequent in PVNS, especially in its diffuse form. 5 9 Therefore, we must consider PVNS a differential diagnosis in atraumatic rotator cuff tears with exuberant joint effusion, 9 and in patients with adhesive capsulitis or any other inflammatory process with a slower course.

The radiographic changes are not very specific. In the initial stages, radiographical findings are unremarkable, as in the case herein reported. In more advanced stages, bone erosion and subchondral cysts are evident, 1 especially in the humerus. 5 Calcifications are uncommon. 1 11

The gold standard for PVNS diagnosis and postoperative follow-up is MRI 1 4 ( Fig. 4A-B ). In the case herein reported, T1- and T2-weighted images showed a lesion with a hypointense signal, a diffuse infiltrate in soft tissues, and hemosiderin deposits resulting in signal reduction ( Fig. 1A-C ). More advanced cases feature synovial membrane thickening, occasional bone erosion, and tendinous and ligament alterations. Still, PVNS is not very specific, being often confused with RA or soft tissue sarcoma. 3

The localized and diffuse forms have the same histological characteristics but different biological behavior, treatment, and prognosis; the diffuse form is more aggressive. 1 Histologically, PVNS consists of a mixture of cells in different proportions. There are two types of mononuclear cells: a small one (histiocyte-like), the main neoplastic cell, and larger cells with amphophilic cytoplasm and lobulated or kidney-shaped nucleus together with multinucleated giant cells (osteoclast-like) and macrophages loaded with hemosiderin, 1 as revealed in the histopathological analysis of the surgical specimen ( Fig. 3 ).

**Fig. 6:**
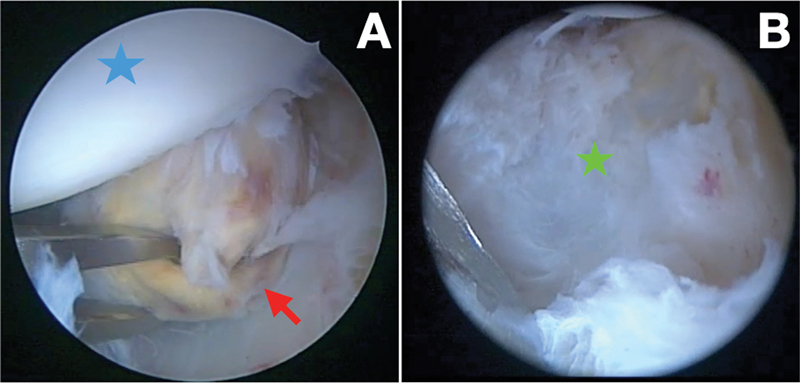
Arthroscopic appearance of the PVNS in the anteroinferior region of the left shoulder. ( **A** ) Superior portal view with arthroscopic forceps in the posterior portal holding the lesion. The lesion was yellowish due to hemosiderin deposits (red arrow). Humeral head (blue star). ( **B** ) Superior portal view. Anteroinferior joint capsule of the shoulder after marginal resection of the lesion and synovectomy (green star).

The treatment of choice consists of complete removal of all pathological tissue and total synovectomy. For the localized form, the prognosis is better, because the resection tends to be total, resulting in a low recurrence rate 3 ( Fig. 6A-B ).

The arthroscopic treatment yields better functional outcomes, facilitating a more effective synovectomy and resulting in fewer complications than those of open surgery. 2 10 Rotator cuff and joint cartilage involvement must be assessed and performance of debridement or repair of the tendon lesion may be required. Arthroplasty is indicated in cases of joint destruction. 5

## Adjuvant Treatments

Radiotherapy as an adjuvant treatment to synovectomy seems to decrease the local recurrence rate of diffuse PVNS. However, there is no consensus regarding this approach if satisfactory surgical resection is feasible. The literature often indicates radiotherapy when total synovectomy and complete resection of extra-articular lesions is not possible. However, some studies discuss the long-term results of this strategy alone. 12 13 14 15 As such, we understand that the treatment must be tailored to each patient.

Regarding pharmacological therapy, ongoing research addresses monoclonal antibodies and tyrosine kinase inhibitors. 16 The role and effectiveness of these medications remain unclear due to the limited number of high-quality studies and some contradictory results. 17 Thus, they are often used for diffuse and refractory cases, which would probably not benefit from surgical intervention. 18

Surgical (arthroscopic, open, or both) resection still is the therapeutic mainstay for PVNS in most patients. However, there is a growing role for adjuvant therapies, especially in cases of diffuse or recurrent disease, but understanding their risks and benefits requires further studies. 17

The case herein reported is unusual, and we call attention to the differential diagnosis of adhesive capsulitis. We emphasize the importance of requesting an MRI scan for patients with refractory capsulitis to exclude or identify other lesions, such as PVNS. Arthroscopic resection of the lesion and synovectomy resulted in excellent functional outcomes during a follow-up longer than 4 years, with no recurrence observed on imaging tests.
