# Supplementary material for: Localized Pigmented Villonodular Synovitis in the Shoulder: Report of a Case Treated through Arthroscopy
Source: Rev Bras Ortop (Sao Paulo). 2024 Apr 22;59(Suppl 2):e188–93. doi: 10.1055/s-0044-1779313 (PMC11679623; doi:10.1055/s-0044-1779313)
Supplement: Related texts

# Research article: Forma localizada da sinovite vilonodular pigmentada no ombro: Relato de caso tratado por artroscopia

**Keywords:** articulação do ombro, artroscopia, lesões do ombro, tumor de células gigantes de bainha tendinosa

## Abstract

A sinovite vilonodular pigmentada (SVNP), na forma localizada no ombro, é rara e pouco descrita na literatura. Apresentamos o caso de uma paciente de 58 anos, do sexo feminino, sem relato de trauma, com sintomas álgicos de 2 meses de evolução, sem irradiação para o membro, associados a perda de força de elevação e limitação progressiva da mobilidade ativa e passiva. A paciente realizou fisioterapia sem supervisão, sem melhora dos sintomas. A ressonância magnética (RM) do ombro mostrou imagem ovalada com aproximadamente 2 cm de diâmetro na região anteroinferior da articulação glenoumeral com focos de hipossinal em seu interior. Realizamos o tratamento artroscópico, com ressecção marginal da lesão e tenotomia da cabeça longa do bíceps. O resultado do exame anatomopatológico constatou SVNP. Após 4 anos e 5 meses da cirurgia, a paciente encontra-se sem dor, e apresenta recuperação total da função do ombro esquerdo, com RM de controle sem recidiva, o que demonstra a eficácia do tratamento artroscópico neste caso.

## Introdução

A sinovite vilonodular pigmentada (SVNP), também chamada de tumor tenossinovial de células gigantes, é uma doença proliferativa que acomete a membrana sinovial nas articulações, bursas ou bainhas tendinosas, e que está associada ao acúmulo de hemossiderrina. 1 É um tumor benigno raro, altamente proliferativo e localmente agressivo, que destrói a articulação, 2 apresenta altas taxas de recidiva, mas raramente ocorre transformação maligna. 1 3 A etiologia é desconhecida, 2 com possível associação a um trauma prévio e sangramento articular, 1 ou artrite reumatoide (AR). 4 O diagnóstico diferencial é o de outras artrites, como AR, artropatia hemofílica, tuberculose e neoplasias. 4 A prevalência é estimada em 1,8 casos por 1 milhão de pessoas, e a doença acomete adultos jovens entre a terceira e a quarta décadas de vida, sem preferência por sexo, 5 6 7 mas também pode ser encontrada em crianças. 4

A SNVP costuma ser mono, intra ou extra-articular, 1 e o envolvimento poliarticular é excepcionalmente raro. 6 Pode se manifestar na forma localizada (massa tumoral única) ou, mais frequentemente, em sua forma difusa, quando afeta o compartimento articular ou toda a membrana sinovial da articulação envolvida. 1 3 O envolvimento do ombro é bem raro; 5 8 9 10 Mahieu et al. 8 observaram que até o ano de 2001 apenas 30 casos de SVNP de ombro haviam sido descritos na literatura nas línguas francesa e inglesa, e estimaram que sua prevalência nesta articulação deva ser menor do que 2%. 5 8 9

## Relato de Caso

Uma paciente do sexo feminino, de 58 anos, com dor no ombro esquerdo havia 2 meses, com perda de força de elevação e piora progressiva, sem história de trauma, sem irradiação para o braço, havia realizado fisioterapia sem melhora dos sintomas. Ela apresentava histórico de fibromialgia, distúrbio da ansiedade, e capsulite adesiva no ombro contralateral. Ao exame físico, observou-se ectoscopia sem alterações, mobilidade articular com limitação apenas da rotação lateral (arco de movimento ativo em elevação, rotação lateral e rotação medial de 150°, 70° e T10 no ombro direito, e de 150°, 50° e T10 no ombro esquerdo, respectivamente), força de elevação de grau 4 (teste de Jobe positivo) e manobras irritativas positivas para impacto subacromial (testes de Neer e de Hawkins positivos ) e para inflamação do tendão da cabeça longa do bíceps (testes de Speed e de Yergason positivos). As radiografias não apresentavam alterações, e um exame de ressonância magnética (RM) mostrou uma imagem ovalada com 2 cm de diâmetro na região anteroinferior da articulação glenoumeral com focos de hipossinal em seu interior ( Fig. 1 ).

**Fig. 1:** Exames de ressonância. ( **A** ) Cortes sagitais, mostrando imagem oval junto à borda inferior do tendão subescapular, se projetando para dentro da articulação glenoumeral (seta). Nota-se sinal hipodenso da lesão. ( **B,C** ) O exame após a injeção de contraste nos cortes axial e coronal do ombro esquerdo, respectivamente, evidenciando imagem ovalada com aproximadamente 2 cm de diâmetro na região anteroinferior da articulação do ombro, (setas) com focos de hipossinal em seu interior.

A paciente foi tratada com ressecção marginal da lesão e tenotomia da cabeça longa do bíceps por via artroscópica em decúbito lateral. Com a câmera no portal posterior (alternando óticas de 30° e 70°), a ressecção foi realizada através do portal anterior, feito um pouco mais medial para se ter acesso ao colo do úmero. Na cirurgia identificamos uma massa marrom- amarelada, fibrótica, nodular, pediculada na região anteroinferior da articulação, com o pedículo oriundo da inserção capsular no colo do úmero, próximo ao recesso axilar ( Fig. 2 ). A ressecção foi realizada com pinças artroscópicas, e a sinovectomia no recesso axilar foi realizada sem aspiração, para evitar lesão do nervo axilar. A peça cirúrgica foi encaminhada para exame anatomopatológico, no qual se confirmou o diagnóstico de SNVP ( Fig. 3 ).

**Fig. 2:** Aspecto nodular com pigmentação por depósitos de hemossiderina, que conferem uma coloração marrom-amarelada. Peça retirada por ressecção marginal artroscópica.

**Fig. 3:** Sinovite vilonodular pigmentada, constituída por células redondas e ovaladas sem atipias, semelhantes a histiócitos (seta amarela), macrófagos com depósitos de hemossiderina (seta preta), com permeio de células gigantes (seta azul), sobre o estroma fibroconjuntivo. Abreviatura: HE, hematoxilina-eosina.

Não realizamos terapias adjuvantes; iniciamos fisioterapia analgésica e motora na primeira semana após a cirurgia, e a paciente evoluiu inicialmente com dor e rigidez articular, que melhorou gradualmente nos dez meses seguintes. A primeira RM de controle pós-operatório foi realizada após seis meses, e mostrava sinais de espessamento capsular compatível com capsulite adesiva. Já a segunda RM, realizada após um ano e nove meses da cirurgia, não mostrou imagens sugestivas de capsulite adesiva, nem sinais de recidiva da doença neoplásica ( Fig. 4 ). Hoje, passados 4 anos e 5 meses da cirurgia, a paciente relata estar assintomática, fazendo atividade física, e apresenta recuperação total da função do ombro operado (ombro esquerdo), com boa mobilidade ativa (arco de movimento ativo em elevação, rotação lateral e rotação medial de 150°, 70°, e T10 no ombro direito, e de 150°, 60° e T10 no ombro esquerdo, respectivamente) ( Fig. 5 ), força de elevação de grau 5, sem dor (teste de Jobe negativo), manobras irritativas negativas para impacto subacromial (testes de Neer e Hawkins negativos) e manobras negativas para inflamação do tendão da cabeça longa do bíceps (testes de Speed e de Yergason negativos).

**Fig. 4:** ( **A,B** ) Exame de RM nos cortes sagital e axial do ombro esquerdo solicitado para controle pós-operatório após 1 ano e 10 meses. Não foram observado imagens sugestivas de recidiva da doença.

**Fig. 5:** Aspecto clínico pós-operatório após 4 anos e 5 meses do procedimento cirúrgico.

A paciente deste relato de caso deu o seu consentimento verbal e escrito, e autorizou a apresentação das suas informações de saúde.

## Discussão

A SVNP no ombro é uma doença rara, invasiva e agressiva localmente, e pode causar grave morbidade articular. Para o diagnóstico, é necessário correlacionar os achados clínicos, os exames de imagem, e as análises histológicas. 3 O tratamento artroscópico é uma alternativa minimamente invasiva e eficaz para a ressecção marginal dessas lesões, especialmente para um caso como este, no qual a lesão estava localizada no recesso axilar.

Os sintomas são inespecíficos, o que torna o diagnóstico difícil. Normalmente ocorre artralgia com limitação antálgica progressiva dos movimentos, 9 11 rigidez articular, 11 eritema, derrame articular e edema no ombro. 3 9 No caso aqui relatado, inicialmente, a hipótese era a de capsulite adesiva com base na dor, na rigidez apresentada no exame físico inicial, e no histórico de capsulite do ombro contralateral. Porém, a RM mostrou imagem sugestiva de SVNP na forma localizada, cujos sintomas geralmente são mecânicos, 4 decorrentes da interposição da massa tumoral no espaço articular.

Rupturas extensas do manguito rotador, com osteoartrite e erosão óssea associada, são comuns, sobretudo em sua forma difusa. 5 9 Sendo assim, é importante considerar a SVNP como diagnóstico diferencial nas rupturas atraumáticas do manguito rotador associadas a um exuberante derrame articular, 9 assim como na capsulite adesiva ou qualquer outro processo inflamatório de curso mais lento.

As alterações radiográficas são pouco específicas. Nas fases iniciais, as radiografias são normais, como no caso aqui relatado; já em fases mais avançadas, evidencia-se erosão óssea e cistos subcondrais, 1 sobretudo no úmero. 5 Calcificações não são comuns. 1 11

A RM é o padrão-ouro para o diagnóstico da SVNP e para o acompanhamento e controle pós-operatórios 1 4 ( Figs. 4A-B ). No caso aqui relatado, as imagens ponderadas em T1 e T2 mostraram lesão com sinal hipodenso, e infiltrado difuso envolvendo tecidos moles, com os depósitos de hemossiderina que causam redução do sinal tanto nas imagens em T1 quanto nas em T2(3) ( Figs. 1A-C ). Em casos mais avançados, ocorre o espessamento da membrana sinovial, e eventuais erosões ósseas, e alterações tendíneas e ligamentares. Ainda assim, a SVNP é pouco específica, e muitas vezes é confundida com AR ou sarcoma de partes moles. 3

As formas localizada e difusa têm as mesmas características histológicas, mas comportamento biológico, tratamento e prognósticos diferentes, sendo a forma difusa mais agressiva. 1 Histologicamente, a SVNP é composta por uma mistura de células com diferentes proporções. Existem dois tipos de células mononucleares: uma pequena (semelhantes a histiócitos, *histiocyte-like* , em inglês,) que constitui a célula principal do tumor, e outras células maiores com citoplasma anfofílico e núcleo lobulado ou em forma de rim, e que são acompanhadas por células gigantes (semelhantes a osteoclastos, *osteoclast-like* , em inglês) multinucleadas, além de macrófagos carregados de hemossiderina, 1 como visto na análise da peça ( Fig. 3 ).

A remoção completa de todo tecido patológico e sinovectomia total são o tratamento de escolha. Na forma localizada, o prognóstico é melhor, pois a ressecção tende a ser completa, o que resulta em baixa taxa de recidiva 3 ( Figs. 6A-B ).

**Fig. 6:** Aspecto artroscópico da SVNP localizada na região anteroinferior do ombro esquerdo. ( **A** ) Visão pelo portal superior com pinça artroscópica posicionada no portal posterior, segurando a lesão. Lesão localizada, de coloração amarelada e pontos de pigmentação por depósitos de hemosiderina (seta vermelha). Cabeça umeral (estrela azul). ( **B** ) Visão através do portal superior: cápsula articular anteroinferior do ombro após ressecção marginal da lesão localizada e sinovectomia (estrela verde).

O tratamento artroscópico oferece melhores resultados funcionais, e facilita uma sinovectomia mais eficaz, com menores complicações quando comparadas às da cirurgia aberta. 2 10 O comprometimento do manguito rotador e da cartilagem articular deve ser avaliado, e pode ser necessário realizar o desbridamento ou o reparo da lesão tendínea. Em casos de destruição articular, a artroplastia está indicada. 5

## Tratamentos Coadjuvantes

A radioterapia usada como tratamento adjuvante à sinovectomia parece diminuir a taxa de recorrência local na forma difusa da doença. No entanto, não há um consenso com relação a essa abordagem quando se consegue realizar uma ressecção cirúrgica satisfatória da lesão. A tendência da literatura parece ser de indicar a radioterapia nos casos em que a sinovectomia total e a ressecção completa das lesões extra-articulares não podem ser feitas, apesar de alguns estudos discutirem os resultados em longo prazo dessa estratégia quando aplicada de forma isolada. 12 13 14 15 Neste contexto, entendemos que cada paciente deve ser manejado de acordo com suas particularidades.

Como terapia farmacológica, existem pesquisas em andamento com anticorpos monoclonais e inibidores de tirosina-quinase. 16 No entanto, devido ao número limitado de estudos de alta qualidade e a alguns resultados contraditórios, o papel e a eficácia dessas medicações ainda não estão claros. 17 Dessa forma, sua utilização tende a ser reservada para os casos de doença difusa e refratária, que provavelmente não se beneficiariam com a intervenção cirúrgica. 18

Para a maioria dos pacientes, a ressecção cirúrgica (pelas vias artroscópica e/ou aberta) continua a ser a base do tratamento para SVNP. No entanto, o papel das terapias adjuvantes é crescente, sobretudo nos casos de doença difusa ou recidivante, mas ainda faltam mais estudos para que se compreenda melhor o seu risco/benefício. 17

O caso relatado é incomum, e alertamos para o diagnóstico diferencial com capsulite adesiva. Ressaltamos a importância de solicitar a RM em casos refratários de capsulite para excluir ou identificar outras lesões, como ocorrido no caso aqui relatado. O resultado funcional da ressecção artroscópica da lesão e da sinovectomia foi ótimo, com seguimento maior do que quatro anos, e sem recidiva nas imagens realizadas.
